# Treatment of Pellagra

**Published:** 1909-10

**Authors:** 


### EDITORIAL DEPARTMENT.

### Treatment of Pellagra.

Much interest has been aroused during the last two years in the subject of pellagra. A study of the disease in the United States has thus far shown that it is widely distributed throughout the South, and present in some localities in the North. The question of prognosis and treatment is naturally, therefore, one of much interest. Dr. C. H. Lavinder, of the Public Health and Marine Hospital Service, who for more than a year has been devoting his time to a study of the disease, has in a recent article* given a brief review of the subject.

He states that the prognosis must invariably be considered as grave, and that complete recovery can seldom be assured. Reliable statistics on the subject in the United States are practically limited to asylum cases, and give a mortality of 67 per cent. It must be borne in mind, however, that asylum cases are undoubtedly the more advanced and hopeless ones, and for that reason will give a mortality much above the average. Lombroso gives statistics of hospital cases in Italy in 1883 and 1884, showing a mortality of 13 per cent, whereas Wollenberg gives Italian statistics for 1905, showing a mortality of a little over 4 per cent. The disease resembles tuberculosis, both in that it is an insidious and chronic condition, and that much depends upon early diagnosis and treatment, prognosis of early cases being far better than advanced ones. The importance of this is apparent when it is considered that the disease is an intoxication, and that it is probably associated with diseased corn or corn products used as food.

Predisposition is believed to be an important factor in this disease. Lowered physical resistance, mental worry, insufficient food, bad housing and alcoholism are supposed to render one more susceptible.

In Italy laws have been passed regulating the use and storing of corn and its derivatives, institutions have been established for the care and treatment of pellagrins; improved agricultural methods are encouraged, and assistance is given to the sick in many ways by the government.†

---

*Public Health Reports, Sept. 10, 1909 Copies of this article can be obtained by making request to the Surgeon-General, Public Health and Marine Hospital Service, Washington, D. C.

†Public Health Reports, July 23, 1909, pp. 1053-1054.

In the treatment of patients Lombroso recommends a liberal diet; in some cases he uses baths and cold douches, believing them to be of benefit in certain cases with nerve and skin manifestations; he has found arsenic a valuable remedy, and sodium chloride of service.

Some authors have reported good results from the use of the newer arsenical preparations atoxyl and soamin.

Transfusion of blood from cured cases to the sick has been tried, and may prove of value.

